# Fanconi Anemia Pathway: Mechanisms of Breast Cancer Predisposition Development and Potential Therapeutic Targets

**DOI:** 10.3389/fcell.2020.00160

**Published:** 2020-04-02

**Authors:** Can-Bin Fang, Hua-Tao Wu, Man-Li Zhang, Jing Liu, Guo-Jun Zhang

**Affiliations:** ^1^Chang Jiang Scholar’s Laboratory/Guangdong Provincial Key Laboratory for Diagnosis and Treatment of Breast Cancer, Shantou University Medical College, Shantou, China; ^2^Department of General Surgery, The First Affiliated Hospital of Shantou University Medical College, Shantou, China; ^3^Department of Physiology, Shantou University Medical College, Shantou, China; ^4^The Cancer Center and the Department of Breast-Thyroid Surgery, Xiang’an Hospital of Xiamen University, School of Medicine, Xiamen University, Xiang’an, China

**Keywords:** breast cancer, Fanconi anemia, susceptibility, SNP, predisposition

## Abstract

The maintenance of genomic stability is crucial for species survival, and its failure is closely associated with tumorigenesis. The Fanconi anemia (FA) pathway, involving 22 identified genes, plays a central role in repairing DNA interstrand cross-links. Importantly, a germline defect in any of these genes can cause Fanconi’s anemia, a heterogeneous genetic disorder, characterized by congenital growth abnormalities, bone marrow failure, and predisposition to cancer. On the other hand, the breast cancer susceptibility genes, *BRCA1* and *BRCA2*, also known as *FANCS* and *FANCD1*, respectively, are involved in the FA pathway; hence, researchers have studied the association between the FA pathway and cancer predisposition. Here, we mainly focused on and systematically reviewed the clinical and mechanistic implications of the predisposition of individuals with abnormalities in the FA pathway to cancer, especially breast cancer.

## Introduction

Fanconi Anemia (FA), a rare autosomal or x-chromosomal recessive human genetic disease, was first described by Guido Fanconi in 1927 (Nalepa and Clapp, 2018), and is characterized by congenital growth abnormalities, bone marrow failure, and predisposition to cancer. During the last 2–3 decades, we have gained remarkable insight into the clinically and biologically complex cancer predisposition syndrome. Although FA occurs rarely (1–5 per million), the heterozygous carriers are present at a much higher frequency (1/300) (D’Andrea, 2010). Biallelic mutations in the genes of the FA pathway reportedly cause FA.

**FIGURE 1:**
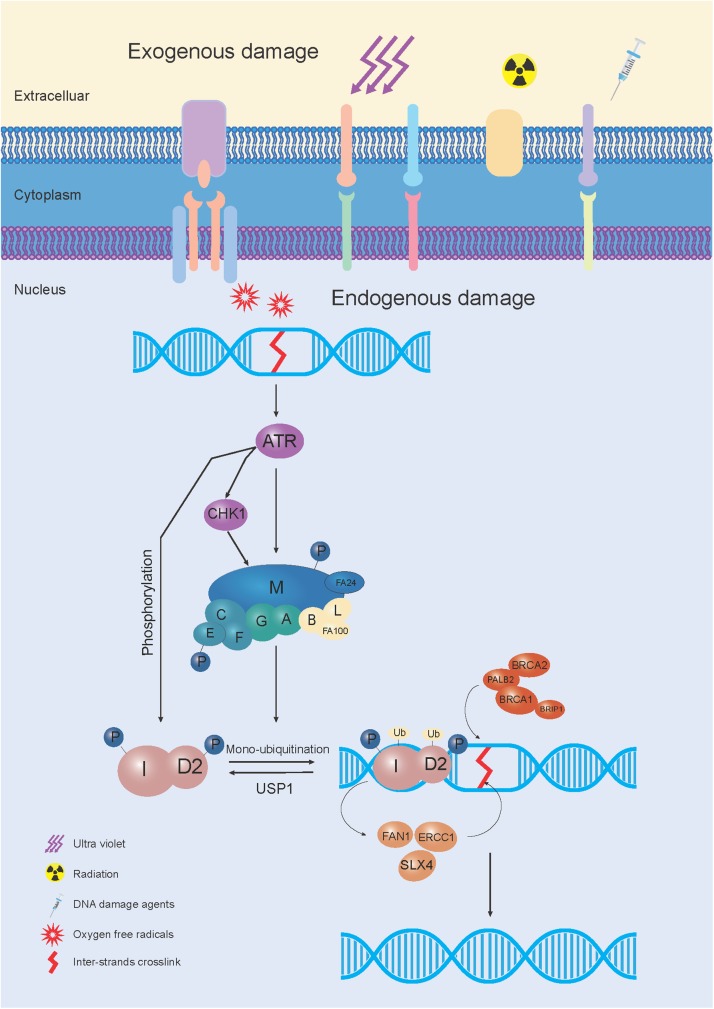
The schematic elucidation of the FA pathway mechanism used during DNA repair. In response to exogenous and/or endogenous damage, 8 FA genes (*FANCA*, *FANCB*, *FANCC*, *FANCE*, *FANCF*, *FANCG*, *FANCL*, and *FANCM*) were assembled into the FA core complex, which functioned as a nuclear E3 ubiquitin ligase complex, to monoubiquitinate the I-D heterodimer. The monoubiquitinated I-D heterodimer was localized to the damaged chromatin, and interacted with DNA-repair proteins and other FA proteins (FANCD1, FANCDN, FANCJ, and FANCS) in the FA pathway, to conduct the repair process through homologous recombination (HR). After the damage was repaired, monoubiquitin was removed from the I-D complex by a de-ubiquitylation enzyme, Ubiquitin Specific Peptidase 1 (USP1), to “turn off” the network.

The FA pathway, also called the FA-BRCA pathway, is a fundamental DNA repair pathway that recognizes DNA damage and orchestrates DNA damage responses, especially for DNA interstrand crosslink (ICL) repair (Su and Huang, 2011). Owing to the functional complementation of ICL sensitive cells, 22 FA or FA-like genes have been identified (Box 1; Knies et al., 2017; Nalepa and Clapp, 2018). Among these, 8 genes (*FANCA, FANCB, FANCC, FANCE, FANCF, FANCG, FANCL*, and *FANCM*) were reported to assemble into a nuclear E3 ubiquitin ligase complex, named the FA core complex, which can monoubiquitinate the FANCD2/FANCI heterodimer (I-D heterodimer). The monoubiquitinated I-D heterodimer localizes to the damaged chromatin, and interacts with DNA-repair proteins and other downstream FA proteins (FANCD1, FANCDN, FANCJ, and FANCS), to perform repair via homologous recombination (HR) (Kim and D’Andrea, 2012). After the repair process is completed, the de-ubiquitylation enzyme, Ubiquitin Specific Peptidase 1 (USP1), removes the monoubiquitin from the I-D complex, to turn off the network, for recycling to be performed (Kim and Kim, 2016; Figure 1).

**Box 1. List of genes in the FA pathway.**

The FA pathway, also called the FA-BRCA pathway, is a fundamental DNA repair pathway, with 22 genes, i.e., *FANCA, FANCB, FANCC, FANCD1, FANCD2, FANCE, FANCF, FANCG, FANCI, FANCJ, FANCL, FANCM, FANCN, FANCO, FANCP, FANCQ, FANCR, FANCS, FANCT, FANCU, FANCV*, and *FANCW.*

A typical cellular feature of cells derived from FA patients is that they are specifically hypersensitive to ICL anti-tumor agents, such as mitomycin C, cisplatin, anddiepoxybutane (D’Andrea and Grompe, 2003), which subsequently increase chromosomal breakage, arrest numerous cells at the G2/M phase, and induce apoptosis (Bhattacharjee and Nandi, 2017). Clinically, even without classical physical findings, the high accumulation of chromosomal breakage products, which occurs during the diepoxybutane chromosome fragility assay, could enable the diagnosis of FA (Auerbach, 2009). More importantly, increased chromosomal breakage predisposes FA patients to cancer. Malignancies develop in about 20% of FA patients with homozygous mutations, such as acute myelogenous leukemia, squamous-cell carcinomas of the head and neck, gynecologic squamous-cell carcinoma, and esophageal carcinoma (Dluhy et al., 2005). Interestingly, heterozygous mutations in FA genes, i.e., *BRCA1/FANCS* and *BRCA2/FANCD1*, confer an increased risk of cancer occurrence, especially breast cancer (Petrucelli et al., 2010). Hence, this article mainly focuses on and systematically reviews the clinical and mechanistic implications of the predisposition of individuals with abnormalities in the FA pathway to cancer, especially breast cancers.

## Fa Pathway and Breast Cancer Predisposition

It is accepted that mutations in the FA pathway are strongly associated with a predisposition to breast cancer (Chen et al., 2014). Representatively, homozygous mutations in *BRCA2* cause a severe form of FA disease (Svojgr et al., 2016). King et al. (2003) found that carriers with inherited heterozygous mutations in *BRCA2* have a high risk for developing breast and ovarian cancer. Similarly, heterozygous *BRCA1* mutations can also cause hereditary breast and ovarian cancer syndromes and the biallelic loss of *BRCA1* genes would cause FA development. Other FA genes, such as *BRIP1/FANCJ* and *PALB2/FANCN*, were also identified as breast cancer susceptibility genes (Seal et al., 2006; Rahman et al., 2007).

Breast cancer is the most common cancer affecting women, and has become the leading cause of cancer-related deaths in females worldwide (Siegel et al., 2018). The incidence of breast cancer are various in different ethnicities, but genetic factors caused by family history influence the occurrence of breast cancer (Brewer et al., 2017). Nevertheless, pathogenic mutations in the breast cancer susceptibility genes *BRCA1* and *BRCA2* only account for 25–40% of familial breast cancers (FBCs) cases (Mahdavi et al., 2019). Another 5–10% FBC cases are attributed to mutations in other rare susceptibility genes, such as *TP53, ATM, PALB2, BRIP1*, and *CHEK2* (Chen and Parmigiani, 2007).

Unsurprisingly, women with inherited pathogenic mutations in *BRCA1* or *BRCA2* have up to an 85% risk of breast cancer development; hence, risk reduction measures, such as intensive radiological screening, prophylactic surgery, or chemoprevention were suggested for these candidates (Thompson and Dixon, 1992). However, the genetic pathogenesis of the major FBC cases remains unknown. Besides *BRCA1* and *BRCA2*, it is extremely important to identify new breast cancer susceptibility genes, for the prevention and treatment of FBCs.

## The Mechanisms of the Fa Pathway Are Associated With the Occurrence of Cancers

DNA repair, an active cellular process that responds to constant DNA damage, is essential for maintaining genomic integrity. Inherited mutations in DNA repair genes were identified to predispose carriers exhibiting genomic instability to cancer. For example, ATM serine/threonine kinase is recruited and activated by DNA double-strand breaks, leading to cell cycle arrest. And the mutations in *ATM* are responsible for the disorder Ataxia telangiectasia (Rotman and Shiloh, 1998). Bloom syndrome protein exhibits both DNA-stimulated ATPase and ATP-dependent DNA helicase activities, and mutations in *BLM* cause Bloom syndrome (Kaneko and Kondo, 2004).

**FIGURE 2:**
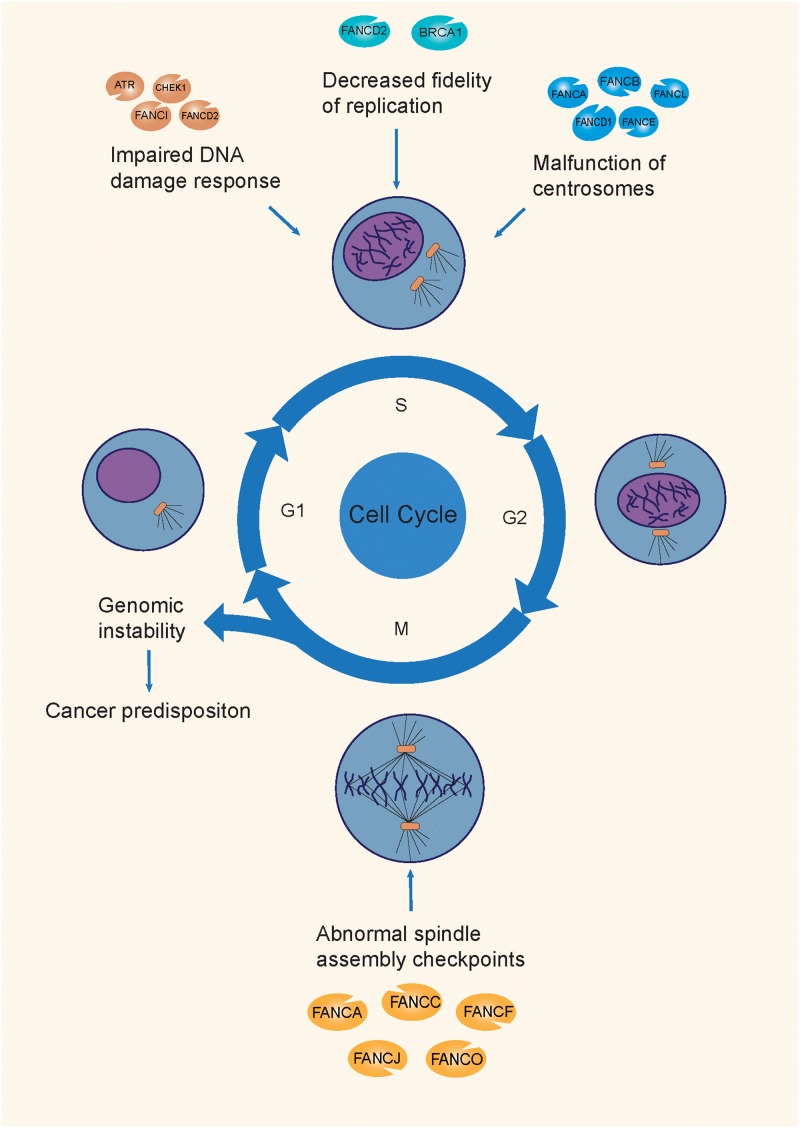
The mechanisms of tumorigenesis attributable to FA mutations. FA genes maintain genomic integrity through the different phases of the cell cycle, by participating in the DDR process, replication fork protection, normal centrosome function, and spindle assembly checkpoints. Mutations on different FA genes are involved in different mechanisms during the cell cycle, causing genomic instability, and causing a predisposition to cancer.

The following section will describe the mechanisms of the FA pathway involved in the repair of the ICL damage, and the corresponding mutations that cause a genomic integrity deficit and promote tumorigenesis (Joenje and Patel, 2001; Figure 2).

### Impaired Interphase DNA Damage Response (DDR)

FA proteins are involved in DDR at multiple levels. First, the DNA damage sensor, ataxia-telangiectasia, and RAD3-related (ATR) kinases, together with its downstream kinase checkpoint kinase 1 (CHK1), detect DNA lesions (mainly stalled replication forks in ICLs), and initiate a response from the FA pathway, by phosphorylating the FA core complex and I-D heterodimer (Ishiai et al., 2017). Subsequently, the activated DDR-stabilized TP53 protein boosts the transcription of cyclin-dependent kinase inhibitor 1A (CDKN1A), to inhibit proliferation and facilitate repair progression (Warfel and El-Deiry, 2013). Meanwhile, the FA core complex monoubiquitinates the I-D heterodimer and promotes ICL repair by causing nucleases, such as FANCP (SLX4), Fanconi-associated nuclease 1 (FAN1), and XPF-ERCC1 to cleave injured DNA strands (Yamamoto et al., 2011; Pizzolato et al., 2015). Finally, the repair process is completed through HR, mainly by the FA downstream genes *FANCS*, *FANCD1*, and *FANCO* (Kim and D’Andrea, 2012). Mutations in such FA genes would impair the DDR process, leading to genomic instability.

### Decreased Replication Fork Protection and Fidelity

Besides the DDR process, Schlacher et al. (2012) reported a novel repair-independent mechanism, that is *FANCD2*- or *BRCA1*-mediated stalling of replication forks, in order to promote the degradation of replication forks and increase replication fidelity, thereby maintaining genomic stability during DNA replication. Normally, BRCA1 interacts with RAD51 during the process of repair of DNA double-strand breaks (Boulton, 2006). The regular level of FANCD2 and FANCM prevented replication fork damage caused by poor coordination between DNA replication and transcription. Surprisingly, in *FANCD2*-deficient cells, increased RAD51 expression levels enhanced the protection of replication forks. Moreover, *FANCD2*-mediated fork protection showed epistatic effects with *RAD51*, which were indicative of an unanticipated fork protection process, and a repair-independent pathway of FA proteins that prevented genomic instability (Schlacher et al., 2012).

### Supernumerary or Over-Replication of Centrosomes

The centrosome serves as the main microtubule-organizing center and regulator of cell cycle progression in animal cells. During mitosis, the nucleated microtubule of the centrosome promotes mitotic spindle assembly, via chromosomal interactions (Nalepa et al., 2013). The presence of extra centrosomes was linked to chromosomal instability, and caused aneuploidy and cancer, by promoting merotelic kinetochore-spindle association (Ganem et al., 2009).

Nalepa et al. (2013) performed RNAi screening and the results showed that 8 FA proteins (FANCA, FANCB, FANCD1, FANCD2, FANCE, FANCG, FANCL, and FANCN) distinctively localized to centrosomes during mitosis, while FANCC and FANCA localized to the mitotic spindle in a cell-cycle dependent manner. Interestingly, an abnormally high number of centrosomes was observed in the primary fibroblasts of FA patients, as compared to that in the controls. Moreover, the accumulation supernumerary centrosomes were observed in artificial FA-deficient cells (Nalepa et al., 2013), suggesting that besides FANCD1, other FA proteins are also essential for maintaining normal centrosome numbers during mitosis. Zou et al. (2014) discovered that in non-stressed Hs587T cells, deficiency of BRCA1 induces centrosome amplication and aneuploidy. However, in hydroxyurea and mitomycin C-treated Hs587T cells experiencing prolonged genotoxic stress, they found that BRCA1 and FANCJ cooperatively promotes DNA damage-induced centrosome amplification (DDICA), by activating polo-like kinase (Zou et al., 2014). On the other hand, in non-invasive breast cancer cell line MCF-7, BRCA1 nuclear export stimulates its regulation of centrosome duplication, which is mediated by the major nuclear export receptor, CRM1 (chromosome region maintenance protein 1) under irradiation treatment (Brodie and Henderson, 2012), predicting the diverse mechanism of BRCA1 function in regulating centrosome amplication in different types of breast cancer cells. Additionally, it was found that cells with deficiencies or dysfunctions of in FA genes promoted error-prone mitosis, along with chromosome missegregation and interphase DNA damage (Abdul-Sater et al., 2015), which contributed to genomic instability, and subsequently, to tumorigenesis.

### Abnormal Spindle Assembly Checkpoint (SAC)

It is known that the capture of the kinetochore by the spindle is a critical step for correct segregation during mitosis, and SAC prevents the separation of duplicated chromosomes until their proper attachment to the spindle apparatus. The SAC can monitor the interactions between kinetochores and spindle microtubules, and be activated by diverse kinds of defects, such as spindle depolymerization (Li and Murray, 1991), dicentric chromosomes (Neff and Burke, 1992), aberrant segregation of centromeres (Wells and Murray, 1996), dysfunctions of kinetochores (Wang and Burke, 1995), or mutations in centromeric DNA (Wang and Burke, 1995), resulting in anaphase arrest, via the inhibition of the anaphase-promoting complex. Hence, the misfunctioning of the SAC can lead to chromosome missegregation, aneuploidy, and even tumorigenesis (Musacchio and Salmon, 2007).

The localization of FA proteins to the mitotic spindle in a cell cycle-dependent manner reveals that FA signaling is essential for the SAC during cell division (Nalepa et al., 2013). It was reported that multiple FA proteins (FANCA, FANCB, FANCC, FANCE, FANCF, FANCD2, FANCI, FANCL, FANCJ, FANCO, and FANCP) are essential for the normal functioning of the SAC (London and Biggins, 2014). Deficiencies in such FA proteins may weaken the functions of SAC, subsequently resulting in genomic instability.

Using all the above mechanisms, the mutations in FA genes finally resulted in genomic instability and subsequent tumorigenesis, however, it is still unclear why germline mutations in certain FA genes lead to tissue-specific tumors. Despite its critical role in ICL repair, the loss of the *BRCA1* function affected specific tissues in the breast and ovaries (Rebbeck et al., 2015). The BRCA1 suppressor hypothesis was put forward, stating that these particular tissues had unique genetic factors or special physiological environments that enhanced cell survival in the absence of *BRCA1*, such as those resulting from the expression of estrogen or other hormones targeting the breast and ovaries. Upon exhibiting additional survival-promoting genetic changes, the cells would be transformed into a malignant tumor (Elledge and Amon, 2002).

## Fa Genes and Their Association With Breast Cancer Predisposition

Based on the mentioned mechanisms, certain FA genes have been identified as breast cancer susceptibility genes, while further evidence is needed to identify others such potential genes (Table 1).

**TABLE 1:** Classification of FA genes that confer to breast cancer susceptibility.

FA gene	Alias	Estimated frequency in FA	Chromosomal location	Molecular function	Breast cancer susceptibility	References
*FANCS*	*BRCA1*	Rare	17q21.31	DNA repair via homologous recombination	Identified	Hall et al., 1990; Miki et al., 1994; Wooster et al., 1994, 1995
*FANCD1*	*BRCA2*	Rare	13q12–13	• DNA repair control and effector recruitment; • Regulates RAD51	Identified	Hall et al., 1990; Miki et al., 1994; Wooster et al., 1994, 1995
*FANCJ*	*BRIP1*	<2%	17q22–24	• 5′-to-3′ DNA helicase; • Binds BRCA1; • Phosphorylated following DNA damage	Identified	Guenard et al., 2008; Ouhtit et al., 2016
*FANCN*	*PALB2*	About 2%	16p12.1	Partner for BRCA2 stability and nuclear localization	Identified	Southey et al., 2010; Blanco et al., 2013; Foo et al., 2017
*FANCO*	*RAD51C*	Rare	17q22	DNA repair via homologous recombination	Identified	Meindl et al., 2010
*FANCM*		<0.2%	14q21.3	• FA core complex assembly • DNA helicase involved in repair of Holliday junctions and replication forks • Recruits the BLM helicase during the DDR	Potential	Kiiski et al., 2014; Peterlongo et al., 2015; Neidhardt et al., 2017
*FANCC*		10%	9q22.3	FA core complex assembly	Potential	Thompson et al., 2012
*FANCD2*		About 2%	3q25.3	• FA I-D complex assembly • Monoubiquitylate and phosphorylate following DNA damage	Potential	Barroso et al., 2006; van der Groep et al., 2008; Mantere et al., 2017
*FANCP*	*SLX4*	Rare	16p13.3	• Resolution of Holliday junctions • Interacts with several nucleases, including FANCQ	Potential	Landwehr et al., 2011; Surowy et al., 2018

### Identified Breast Cancer Susceptibility Genes in the FA Pathway

#### BRCA1/FANCS and BRCA2/FANCD1

**FIGURE 3:**
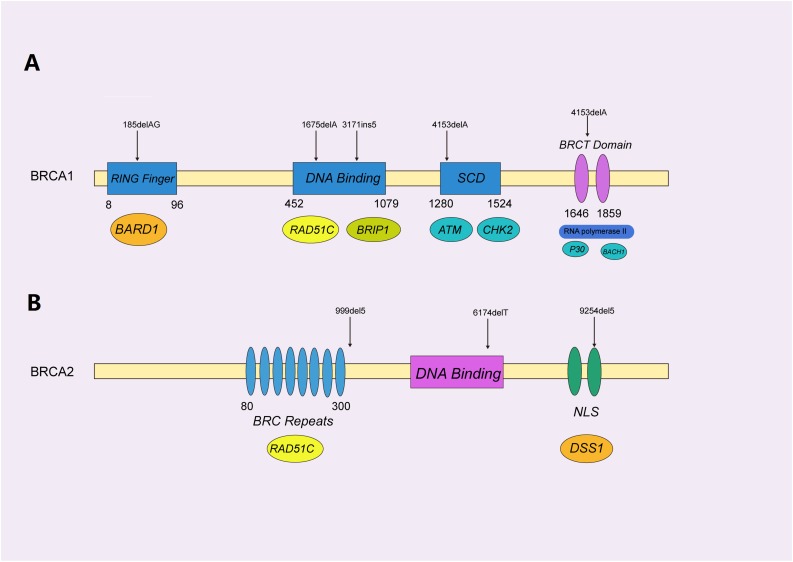
Functional domains of BRCA1/2 protein with pathogenic mutations. **(A)** The functional domains of the BRCA1 protein, mainly containing the RING-finger, SQ-cluster, and BRCT (middle) domains, functionally interacted with BARD1, RAD51C, and ATM (beneath), to orchestrate homologous recombination. The selected reported pathogenic mutations are indicated with black arrows, as shown. **(B)** BRCA2 was represented by a similar schematic figure with different functional domains (middle) and binding partners (beneath); confirmed pathogenic mutations are also shown above.

The human breast cancer type1 susceptibility protein (*BRCA1*) (FA alias *FANCS*) and breast cancer type 2 susceptibility protein *(BRCA2)* (FA alias *FANCD1*) are the most important hereditary breast cancer genes, as identified by linkage studies in 1994 and 1995, respectively (Hall et al., 1990; Miki et al., 1994; Wooster et al., 1994, 1995). *BRCA1* and *BRCA2* are essentially tumor suppressor genes, which mainly help to repair damaged DNA or destroy cells if DNA cannot be repaired, thereby ensuring genomic stability (Gudmundsdottir and Ashworth, 2006). Taken together, mutations in *BRCA1/2* account for 25–40% of FBCs (Antoniou et al., 2001), and up to 10% of all breast cancers (Pfeffer et al., 2017) (Figure 3). Deleterious variants in *BRCA1/2* confer a strong predisposition to breast cancer, and increase the relative risk to carriers by about 10- to 20-fold, as compared to that for the general population (Stratton and Rahman, 2008). During their lifetime, breast cancer carriers have a breast cancer developmental risk of up to 50 and 80% at 70 and 90 years (Chen and Parmigiani, 2007). Besides breast cancer, a dysfunction in *BRCA1/2* is also proven to be associated with an elevated risk of occurrence of other cancers, such as ovarian, pancreatic, prostate, and stomach cancers (Roy et al., 2011). Although the frequencies of *BRCA1/2* mutations vary significantly in different populations, based on geographic regions and ethnicities (Fackenthal and Olopade, 2007), they tend to occur infrequently in most populations; hence, *BRCA1/2* genes are classified as rare high-penetrance breast cancer susceptibility genes (Stratton and Rahman, 2008).

*BRCA1*, encoded by the *BRCA1* gene on 17q21, contains four major domains, i.e., a zinc ring finger (RING) domain, BRCA1 serine cluster domain (SCD), and two BRCA1 C Terminus (BRCT) domains (Rosen et al., 2003). *BRCA1* is mainly involved in repairing double-stranded breaks in DNA and cell cycle checkpoint activation (Caestecker and Van de Walle, 2013), along with transcriptional regulation and chromatin modification (Venkitaraman, 2002; Yoshida and Miki, 2004). Hundreds of mutations have been identified in *BRCA1*, and most of the disease-causing variants of *BRCA1* are present in the BRCT and RING domains, which are essential for the repair function (Nelson and Holt, 2010).

BRCA2 protein, encoded by the *BRCA2* gene on 13q12.3, is responsible for repairing DNA via the specific regulation of the HR pathway, and has a significantly different structure, as compared to that of *BRCA1* (Orelli and Bishop, 2001). It mainly contains eight BRC repeats and the BRCA2 DNA-binding domain, which includes a helical domain (H), three oligonucleotide binding (OB) folds and a tower domain (T) (Roy et al., 2011). Not surprisingly, different mutations in *BRCA1/2* cause variant subtypes of breast cancers. It was reported that pathogenic mutations in *BRCA1* normally result in triple-negative breast cancers (TNBC) (Lee, 2008), while *BRCA2* mutations typically cause the development of ER + luminal subtypes exhibiting a slow proliferation and low level of aggression (Talens et al., 2017).

However, the tumor suppressor mechanism of *BRCA1* and *BRCA2* was thought to have no association with the FA pathway, until Howlett et al. (2002) identified the *FANCD1* gene as *BRCA2* in 2002. The study was based on the fact that cell lines homozygous for *BRCA1*/*2* mutations are hypersensitive to mitomycin-C (Moynahan et al., 2001) and that homozygous *BRCA2* mutant mice have phenotypic features similar to those observed in the mice with FA (Connor et al., 1997). These findings urged Howlett et al. (2002) to screen mutations within *BRCA1*/*2* in FA patients without mutations in known FA genes. They found that they were heterozygous for truncating *BRCA2* mutations in one FA-B and two unassigned FA cases. Moreover, the reference FA-D1 cell line was homozygous for a *BRCA2* splicing mutation that resulted in an in-frame deletion of four amino acids, and an additional FA-D1 patient carried two truncating *BRCA2* mutations (Howlett et al., 2002). All these findings strongly suggested that *BRCA2* caused FA-D1. This surprising but significant discovery not only enabled us to determine an important connection between the FA genes and breast cancer, but also provided an attractive model for identifying more breast cancer susceptibility genes and exploring their underlying mechanism. Thus, a new role of the FA pathway in breast cancer predisposition was discovered.

Similarly, *BRCA1* was identified as FANCS by Sawyer et al. (2015), with a missense mutation in the C terminal, and a frameshift mutation in exon 11 in a 23-year-old female with breast cancer exhibiting multiple congenital abnormalities and an FA-like presentation. Together with a preceding report describing the biallelic loss of *BRCA1* in a young ovarian cancer patient with multiple congenital abnormalities (Domchek et al., 2013), this proof contributed to the identification of *BRCA1* as *FANCS*.

#### BRIP1/FANCJ

*BRIP1* is a member of the RecQ DEAH helicase family, and is encoded by *BRIP1*, a tumor suppressor gene involved in the DNA repair pathway, via its interaction with *BRCA1* (Ouhtit et al., 2016). In Levitus et al. (2004) reported 2 new genetic subtypes excluded from 9 known subtypes (A, B, C, D1, D2, E, F, G, and L), including FA-J, based on 8 unrelated FA patients, and defined FA-J cell line with mono-ubiquitinated FANCD2, which complemented group FA-I but did not complement each other, indicating a downstream defect in FA-J cells (EUFA1289 cells). However, they did not identified the genes defective in complementation groups FA-I nor FA-J. Levitus et al. (2005) successfully identified *BRIP1* as an FA gene in a sub-group of mutated FA-J patients, named *FANCJ* A recurrent nonsense mutation 2392C→T/R798X was identified in 4 individuals with different ethnic backgrounds, as well as three splice mutations in the intervening sequence (IVS), i.e., IVS3 + 5G→T, IVS17 + 2insT, and IVS11-498A→T, which demonstrated the relationship between *BRIP1* and the onset and development of FA-J.

Soon, Seal et al. (2006) identified *BRIP1* as a breast cancer susceptibility gene by detecting several truncating mutations in *BRIP1* that were associated with the onset of breast cancer in high-risk families without mutations in *BRCA1/2*. Further studies illustrated that *BRIP1* was a low/moderate-penetrance breast cancer susceptibility gene (Guenard et al., 2008). Several other mutations, such as C47G/rs4988351, 2971C > G/Q944E/, rs7213430, and rs4986764 (49-51) were reported to cause the elevated risk of breast cancer in different populations, supported the role of *BRIP1* in breast cancer development.

#### PALB2/FANCN

PALB2 was first identified as an interactor of BRCA2 in the DDR process (Xia et al., 2006). PALB2, which is physically bound to BRCA1/2, forms a BRCA complex and maintains genomic integrity via the FA and HR pathways (Sy et al., 2009; Zhang et al., 2009a, b). Unsurprisingly, it was found that mutations in *PALB2* could cause the appearance of FA subtype N, i.e., *FANCN*, presented with skin, thumb, heart and kidney abnormalities and growth retardation like other FA subtype, however, the presention of FANCN patients is similar to the phenotype of biallelic BRCA2 mutations and differs from other FA subtypes, most notably with respect to the high risks of childhood solid tumors, particularly Wilms tumor and medulloblastoma (Reid et al., 2007; Xia et al., 2007). So, the mutations in *PALB2* normally not only resulted in typical FA phenotypes, but also increased the occurrence of pediatric malignancies, Wilm’s tumors, and medulloblastomas (Reid et al., 2007). Importantly, the cancer spectrum caused by mutations in *PALB2* is quite similar to that induced by mutations in *BRCA2*, thereby validating the direct interaction between *PALB2* and *BRCA2* (Nepomuceno et al., 2017).

Almost simultaneously, Rahman et al. (2007) first reported that *PALB2* is a breast cancer susceptibility gene, thereby establishing the fact that mutations in *PALB2* cause a predisposition to breast cancer. Monoallelic truncating *PALB2* mutations were identified in 10/923 individuals with FBCs, conferring a 2.3-fold higher risk for breast cancer, as compared to 0/1,084 controls (Rahman et al., 2007). However, the penetrance of mutations in *PALB2* varied significantly in different populations, ranging in a 2–30-fold higher risk, as compared to that in non-carriers (Southey et al., 2010; Antoniou et al., 2014; Slavin et al., 2017). Several missense variants with an unknown significance have also reportedly been associated with breast cancer (Blanco et al., 2013; Damiola et al., 2015; Li et al., 2015; Nakagomi et al., 2016), among which L35P was identified as the pathogenic variant in a family with a strong history of breast cancer (Foo et al., 2017).

#### RAD51C/FANCO

*RAD51C*, which belongs to the *RAD51* family, is crucial for maintaining genome stability in the HR pathway by binding to single-stranded DNA and unwinding duplex DNA, and forming helical nucleoprotein filaments at the DNA breakage site (Suwaki et al., 2011). As biallelic germline mutations in *RAD51C* were associated with an FA-like syndrome, in 2010, *RAD51C* was demonstrated to be the same as *FANCO* in the FA pathway (Vaz et al., 2010). Meindl et al. (2010) discovered *RAD51C* to be a cancer susceptibility gene, and discovered 6 pathogenic mutations in 1,100 families with breast/ovarian cancer, and not discovering it either in 620 pedigrees with breast cancer alone, or in 2,912 healthy controls. Interestingly, the penetrance level of *RAD51C* is similar to that in BRCA1/2, indicating the important cellular function of *RAD51C* as a tumor suppressor gene in the DNA repair process (Meindl et al., 2010). Osorio et al. (2012) screened the mutations in the *RAD51C* gene in a large series of 785 Spanish families with breast and/or ovarian cancer, and identified that 1.3% exhibited mutations, thus supporting the fact that *RAD51C* played a role as a susceptibility gene.

### Potential Breast Cancer Susceptibility Genes in the FA Pathway

#### FANCM

*FANCM* is the most conserved protein in the FA pathway, and plays an important role in promoting branch migration in Holliday junctions and DNA repair structures at replication forks (Blackford et al., 2012). With its translocase and endonuclease activities, *FANCM* functions as a tumor suppressor gene, by suppressing spontaneous sister chromatid exchanges and maintaining chromosomal stability (Gari et al., 2008). Kiiski et al. (2014) first reported a nonsense mutation in *FANCM*, c.5101C > T (p.Q1701X); it was associated with the breast cancer risk in the Finnish population, and was significantly more frequent among breast cancer patients than in controls, with a particular enrichment observed in TNBC patients. The second variant associated with breast cancer risk, c.5791C > T, was discovered (Peterlongo et al., 2015), followed by several heterozygous loss of function (LoF) mutations in *FANCM* (Neidhardt et al., 2017). All these observations provided evidence that *FANCM* is a candidate breast cancer susceptibility gene.

#### FANCC

The FANCC protein, which is present in the Fanconi anemia complementation group, is involved not only in DNA repair and genome integrity maintenance (Kitao et al., 2006), but also in metabolic disorders (Nepal et al., 2018) and provision of protection against oxidative stress-induced apoptosis (Kulanuwat et al., 2018). In Berwick et al. (2007) found that 6 out of 33 carriers with *FANCC* mutations developed breast cancer, and a 2.4-fold increase in standardized incidence ratios (SIR) was noted among carrier grandmothers. Another three truncating mutations in *FANCC* were observed in 438 breast cancer families, while 1 pathogenic mutation was identified in an additional 957 breast cancer families; no deleterious mutation was reported in 464 healthy controls nor in 1,000 genomic data (Thompson et al., 2012). However, the role of mutations reportedly occurring during breast carcinogenesis remains unclear. Further research is needed, to confirm the possible susceptibility alleles of *FANCC* mutations.

#### FANCD2

As mentioned above, FANCD2 can combine with FANCI to form the I-D heterodimer, which would be monoubiquitinated by E3 ubiquitin ligase; this is regarded as the central step in the activation of the FA pathway (Ishiai et al., 2017). More importantly, it was found that FANCD2 co-localized with BRCA1/2 in DNA damage-inducible foci (Wang et al., 2004; Montes de Oca et al., 2005), suggesting a strong potential association between FANCD2 and FBC. Further studies provided more evidence that established their association. Although Lewis et al. (2005) first reported that there was no evidence regarding the fact that highly penetrant exonic or splice site mutations in *FANCD2* contributed to FBCs, an article published during the next year predicted that mutations in *FANCD2* were associated with an increased risk of sporadic breast cancer (Barroso et al., 2006). Then, van der Groep et al. (2008) discovered that the somatic inactivation of (epi)genetic events in *FANCD2* might be important in both sporadic and hereditary breast carcinogenesis. Rudland et al. (2010) further illustrated that the cytoplasmic loss of *FANCD2* in primary breast carcinomas might allow the selection of cells overexpressing proteins that could induce metastases before surgery. In 2017, truncating mutations in *FANCD2* were discovered, which connected this FA gene with hereditary breast cancer susceptibility during case-control analysis (Mantere et al., 2017), indicating that *FANCD2* is a potential breast cancer susceptibility gene.

#### SLX4/FANCP

SLX4, a DNA repair protein, encoded by the *SLX4* gene, regulates three structure-specific endonucleases (SLX1, XPF-ERCC1, and MUS81-EME1), and is necessary for providing resistance to DNA crosslinking agents, topoisomerase I(TOPI), and poly (ADP-ribose) polymerase (PARP) inhibitors (Shah et al., 2013). Biallelic mutations of *SLX4* (also known as *FANCP*) have been identified in patients with a new subtype of FA, termed as FA-P (Kim et al., 2011; Stoepker et al., 2011). Ponce et al. (2012) detected an *SLX4* missense change, i.e., c.1114C > T (p.Arg372Trp), segregated along with breast cancer genes within the family, which supported the potential role of *SLX4* in conferring breast cancer susceptibility. Even though several studies failed to verify the role of *SLX4* mutations in breast cancer (Landwehr et al., 2011; Bakker et al., 2013; Shah et al., 2013), a potential link between SLX4 and breast cancer predisposition was strongly recommended by Surowy et al. (2018), through the successful identification of a variant rs3810813 in the *SLX4/FANCP* gene, which was significantly associated with both breast cancer and decreased DNA repair capacity.

## Implications for Breast Cancer Therapy

The disruption of the FA pathway results in defective DNA repair, genomic instability, and tumorigenesis, and provides promising targets for breast cancer therapy, by inducing completely different biological characteristics in tumor cells. Strategies for targeting these deficiencies are summarized in the following section.

### Synthetic Lethality and Parp Inhibitors

DNA damage in the human genome mainly involves single-strand breaks (SSBs), double-strand breaks (DSBs), and inter-strand crosslinks. DSBs are restored via double-strand break repair (DSBR), which involves HR and non-homologous end joining (NHEJ). SSBs are restored by single-strand break repair (SSBR), which involves base excision repair (BER), nucleotide excision repair (NER), and mismatch repair (MMR). BER plays the most important role in SSBR, by recruiting other DNA repair players to the site of DNA damage, through the activity of poly (ADP-ribose) polymerases (PARPs) (Caldecott, 2019).

PARPs are involved in various cellular processes, such as DNA repair, DNA replication, recombination, and chromatin remodeling. Among the 17 types of PARPs, PARP-1 plays the most important role during DNA damage, mainly in SSBR (Keung et al., 2019). During SSBR, PARP1 detects the damaged site and binds damaged DNA through its N-terminal zinc finger motifs; then, the catalytic C-terminal domain is activated, to hydrolyze NAD + and produce linear and branched PAR chains, which can extend over hundreds of ADP-ribose molecules (Langelier et al., 2012). Subsequently, several DNA repair proteins, including topoisomerases (TOP), DNA ligase III, DNA polymerase β, and scaffolding proteins, such as X-ray cross-complementing protein 1 (XRCC1) are recruited, to finish the repair process (Rouleau et al., 2010; Palazzo and Ahel, 2018). When PARP-1 is defective or inhibited, SSB cannot be repaired, which results in stalled replication forks and DSBs (O’Neil et al., 2017). In cells exhibiting normal HR, these DSBs can be repaired, to compensate for the loss of PARP1 function. However, in cells exhibiting defective HR, such as breast cancer cells with pathogenic BRCA1/2 mutations, defects cannot be repaired, leading to tumor-specific cell death (Lord and Ashworth, 2017). This is explained by synthetic lethality, originally referring to a lethal phenotype that results from the simultaneous disruption of two genes, while the disruption of either gene alone causes the cell to remain viable (Ashworth and Lord, 2018). Specifically, the simultaneous loss of PARP-1 induced SSBR and BRCA1/2 induced HR would result in cell death, while cells exhibiting a disruption in either of these could survive.

Besides inhibiting PARP catalytic activities, PARP trapping on DNA, a formation of non-covalent protein-DNA adducts was illustrated in the molecular mechanism of the cytotoxicity of PARP inhibitors, considering single-agent activities (Murai et al., 2012). During the repair process, PARP inhibitors effectively induce PARP1 and PARP2 trapping onto DNA and forbid the utilization of NAD + and auto-PARylation, associated with catalytic inhibition of PARylation (Murai et al., 2014). A novel implementation of the proximity ligation assay developed by Hopkins et al. (2019), showed high sensitivity and throughput at single-cell resolution to detect trapped PARP-DNA complexes. Importantly, the toxicity of trapped PARP complexes is not restricted to cancer cells with HR deficiency, but also drive single-agent cytotoxicity in healthy human bone marrow, suggesting the inverse relationship between trapping potency and tolerability (Hopkins et al., 2019). Based on CRISPR screening, a high-confidence set of 73 genes was confirmed to increase PARP inhibitor sensitivity when mutated (Zimmermann et al., 2018). Pommier et al. (2016) systematically reviewed the mechanism of PARP trapping and its relationship with chemoresistance in clinical, provided the implication of PARP trapping for chemotherapy combination. To better understand the two pathways in mediating the cytotoxicity of PARP inhibitors, Wang et al. (2019) designed and constructed a series of small molecule PARP degraders to mimic PARP1 genetic depletion and decouple PARP1 catalytic inhibition from PARP1 trapping, showing promising approaches to suppress PARP1 hyperactivation in various pathological conditions.

Based on the mechanisms for inhibition of PARP catalytic activities and PARP trapping, several PARP inhibitors (PARPi), such as Olaparib (KuDOS/AstraZeneca) (Mateo et al., 2015), Veliparib (Abbvie) (Kummar et al., 2012), Rucaparib (Pfizer/Clovis) (Swisher et al., 2017), and Niraparib (Merck/Tesaro) (Scott, 2017) have been developed and applied in clinical studies. PARPi were particularly effective in the treatment of patients with breast, ovarian, or other cancers, who were *BRCA1* and/or *BRCA2* deficient. For example, Olaparibis the first PARPi approved by the FDA for the treatment of breast cancer patients carrying *BRCA* germline mutations (Tutt et al., 2010). Compared with standard therapy, olaparib monotherapy provided a significant benefit for metastatic breast cancer patients with a germline *BRCA* mutation, with 2.8 months prolonged median progression-free survival (PFS) and 42% reduced risk of disease progression or death (Robson et al., 2017). Recently, a randomized, open-label, phase 3 trial was conducted in advanced breast cancer and a germline *BRCA* mutation to evaluate therapeutic effect of talazoparib, another PARPi, showing the significant benefit of single-agent talazoparib over standard chemotherapy, with respect to 3 months prolonged PFS and 35.4% increased objective response rate (Litton et al., 2018). Apart from *BRCA1/2* mutations, individuals with deficiencies in other FA genes and tumor suppressor genes involved in HR could benefit from the potential therapeutic capacities of PARPi; as the subsequent effects were unclear, they are being investigated (Lord and Ashworth, 2016).

### Hypersensitivity to ICL Agents

Besides synthetic lethality, cells defective in several FA pathway genes, especially those involved in HR, were found to be hypersensitive to certain chemotherapeutic reagents, particularly ICL agents (Van Der Heijden et al., 2003; Chirnomas et al., 2006). Representatively, triple-negative breast cancer patients with germline or somatic pathogenic BRCA1/2 mutations are sensitive to cisplatin or carboplatin, which are recommended as the preferred regiments for HER2-negative breast cancer patients, as per the NCCN Guideline Version 1.2019. It provides an alternative to neoadjuvant chemotherapy or adjuvant chemotherapy treatment in patients with late advanced triple-negative breast cancer. Therefore, researchers have hypothesized that the inactivation of the FA pathway could act as a predictive biomarker of the chemotherapeutic response. Easy and reproducible methods that could be widely adopted for understanding the viability of the pathway need to be developed. Mukhopadhyay et al. (2010) successfully developed a method to determine the HR status by studying RAD51 focus formation in primary cell cultures. The identification of novel agents to which FA pathway-deficient cells are hypersensitive could provide additional therapeutic targets.

In terms of the above two aspects, the FA pathway shows promising clinical implications in cancer therapy. The biochemical mechanisms of the FA pathway need to be studied further, to identify novel biomarkers and develop effective therapeutic targets.

## Discussion

The identified breast cancer susceptibility genes in the FA pathway, including *BRCA1, BRCA2, BRIP1, PALB2*, and *RAD51C*, are essential genes involved in HR, the error-free pathway for DSB repair during physiological cell cycle progression, which repairs replication-associated DNA damage (Michl et al., 2016; Wright et al., 2018). HR is also involved in the final steps of ICL repair, primarily in the S and G2 phase, when a sister chromatid is available as the repair template and provides a high fidelity and error-free solution for repair. Additionally, it is illustrated that deficiencies in the common genes in the FA and HR pathway result in unrepaired DNA damage and sequential genomic instability, and eventually increase the risk of breast cancer and predisposition to certain kinds of cancer (Box 2).

**Box 2. Facts.**

• The germline mutations in the Fanconi anemia pathway partially elucidate the functional basis of genomic instability, predisposition to cancer, and tumorigenesis in diverse human cancers, especially breast cancer.
• The potential underlying mechanisms of the FA pathway involved in tumorigenesis included the impaired interphase DNA damage response, decreased replication fork protection and fidelity, supernumerary or over-replication of centrosomes, and abnormal spindle assembly checkpoints.
• Several FA genes, such as *BRCA1/FANCS*, *BRCA2/FANCD1*, *PALB2/FANCN*, and *RAD51C/FANCO* have been confirmed to be breast carcinoma susceptibility genes at present.

**Box 3. Open questions.**

• Why did heterozygous germline mutations in certain FA genes predisposed carriers to tissue-specific cancers, such as breast cancer?
• Besides the reported susceptibility and potential breast cancer susceptibility genes, are mutations in other FA genes associated with breast cancer, or other types of cancers?
• Besides DNA damage repair, were any other underlying mechanisms involved in the association between FA pathway and breast cancer?
• The search for potential cancer therapy targets and treatment strategies associated with the FA pathway are important research hotspots and have implications in clinical practice.

In summary, the identified susceptibility gene *BRCA2* is required for the loading of *RAD51* onto ssDNA during the repair process (Davies et al., 2001). During HR, *PALB2* (*FANCN*) and *BRIP1* (*FANCJ/BACH1*) functions as the binding partner and regulator for *BRCA1* and *BRCA2*, respectively (Hiom, 2010; Park et al., 2014). On the other hand, the potential breast cancer susceptibility gene in the FA pathway, *FANCM*, is also needed for recruiting CtIP (C-terminal binding protein interacting protein) and MRN (MRE11-RAD50-NBS1) at the site of ICL, during the HR process (Daley et al., 2013). These findings have not only elucidated the crosstalk between the FA and HR pathways, but also provided an insight into the possible mechanism by which mutations in the FA pathway cause a predisposition to breast cancer.

Moreover, other known breast cancer susceptibility genes are either associated with the FA pathway or involved in DNA repair. For example, *ATM*, a rare moderate-penetrance breast cancer susceptibility gene, is responsible for phosphorylation and chromatin recruitment in *FANCM* (Sobeck et al., 2009). *CHEK2*, a serine/threonine kinase, is activated upon DNA damage and implicated in pathways governing DNA repair, cell cycle arrest or apoptosis in response to the initial damage (Apostolou and Papasotiriou, 2017). *TP53* is the most frequent mutational target in human cancers, and mutations in *TP53* are associated with different types of malignancies and adverse prognoses, including during breast cancer (Bellazzo et al., 2018). In conclusion, among all the DNA repair pathways, the FA pathway has the strongest association with increased risk of developing breast cancer. Hence, the FA pathway is also termed as the FA/BRCA pathway.

However, the underlying mechanism remained unclear (Box 3). Is it possible for other FA genes to predispose some specific ethnic group cancer? Why are the roles of *FANCD2* and *FANCI* in cancer predisposition not identified, though they are central participants in the FA pathway? Is it possible for the FA pathway and HR process to be the same, as more and more genes of each are identified to be identical? All these issues still need to be addressed by researchers.

During the past two decades, we have witnessed great advancements in the study of FA, with the identification of more and more FA genes and the biological mechanism of FA was elucidated. It was believed that more and more genes will be identified as FA genes, especially for those involved in HR. This would enable us to gain greater insight into breast cancer susceptibility and the FA pathway, which would provide clinical benefits to patients with FA and breast cancer.

## Author Contributions

JL and G-JZ contributed conception and design of the study. C-BF, H-TW, and JL organized the database, searched the literature, structured, and drafted the manuscript, figures, and table carefully. M-LZ organized the database and drafted the manuscript carefully. JL and G-JZ revised the original manuscript critically. All authors contributed to manuscript revision, read and approved the submitted version.

## Conflict of Interest

The authors declare that the research was conducted in the absence of any commercial or financial relationships that could be construed as a potential conflict of interest.
